# Childbirth Experience Questionnaire (CEQ): research proposal for translation and validation into Sinhala language among a Lankan cohort of women

**DOI:** 10.1186/s13104-019-4499-2

**Published:** 2019-07-25

**Authors:** Malitha Patabendige

**Affiliations:** Obstetrics and Gynaecology, University Obstetrics Unit, North Colombo Teaching Hospital, Ragama, Sri Lanka

**Keywords:** Childbirth Experience Questionnaire, Construct validity, Birth satisfaction, Patient satisfaction, Reliability, Sri Lanka

## Abstract

**Introduction:**

The Childbirth Experience Questionnaire (CEQ) is a Sweden origin, self-administered questionnaire to assess birth satisfaction of women in different aspects of their first labour and birth. It measures four main domains of the woman’s childbirth experience. Those are own capacity, professional support, perceived safety and participation, comprising of 22 items.

**Objectives:**

To conduct a linguistic translation, to conduct a validation study and to assess the psychometric properties of the Sinhala version of the CEQ.

## Introduction

The Childbirth Experience Questionnaire (CEQ) was developed and validated among first-time mothers in 2010 in Sweden [1]. It has been validated in the United Kingdom too in 2015 [2]. It measures four main domains of the childbirth experience. Those are own capacity, professional support, perceived safety and participation comprising of 22 items [1, 2]. This CEQ is a simple self-administered questionnaire to assess women’s satisfaction in different aspects of their first labour and birth [1]. However woman’s perceptions and experiences in childbirth are being rarely evaluated. Moreover, primiparous women are prone to get negative childbirth experiences [1, 2].

Since there is no properly validated instrument in Sinhala language to assess childbirth experience in Sri Lankan women, Sinhala CEQ will be a worthwhile effort. Latest NICE guideline on intrapartum care has also highlighted that any research on the effectiveness of interventions in latent stage of labour needs measuring woman’s satisfaction with childbirth experience [3]. In a similar manner for any study investigating woman’s satisfaction with her birth experience in Sri Lanka needs to have a standardised method like CEQ. Validation of this tool will be important in future research, audit projects as well as in service evaluation. Different tools to assess maternal childbirth experience are essential to identify those who need psychological and moral support. Therefore, this tool can be used to assess various dimensions of childbirth experience in first-time mothers [1].

## Review of literature

The birth of a child is a significant event in a woman’s life and family. Labour room staff should always try to make her labour experience as comfortable and dignified as possible. Although women seems to be satisfied with a healthy baby, the childbirth also has a psychological dimension [4, 5]. Address of these hidden aspects is important for a positive childbirth experience and mother’s long-term well-being [4, 5]. Higher workload in the hospitals resulted a sub-standard level of intrapartum care during childbirth [5]. Studies have reported that mothers often felt alone and unsupported during childbirth process and some of them would not get pregnant again due to the negative experience they had during last childbirth [5]. In the latest Cochrane review on continuous support for women during childbirth has reported that continuous labour support with the help from a labour companion (*doula*) who is a member of the labouring woman’s social network, has been proved to effective in improving laboring women’s satisfaction [6] and hence their childbirth experience. Thus it elaborates the significance of woman’s psychological well-being and achieving a satisfactory childbirth experience in addition to birth of her baby.

The quality of intrapartum care that a particular woman gets has an impact on her physical and emotional well-being [3]. It has both short-term and longer-term effects including care towards the newborn [3]. Good communication with woman, support from labour ward staff and having woman’s wishes respected, can enhance her confidence contributing to make a positive childbirth experience [3]. Negative experiences coming from their first childbirth can increase the risk of postpartum depression. It may give rise to a negative attitude in future pregnancies and childbirth too prompting women to request elective caesarean section [7]. This CEQ may contribute to uplift the quality in maternal health care as in several new strategies which are coming up in Sri Lanka such as WHO Safe Childbirth Checklist [8–10].

## Objectives of the study

General Objective is to translate and validate the CEQ from English language to the cultural context and lifestyle related to the Sinhala language. Specific objectives are to conduct a linguistic translation, to conduct a validation study and to assess the psychometric properties of the Sinhala version of the CEQ.

## Materials and methods

### Study design and setting

A validation study to translate and validate the CEQ from English to Sinhala language. University Unit of Obstetrics and Gynaecology, North Colombo Teaching Hospital (NCTH), Ragama, Sri Lanka.

### Study population

Mothers who have undergone their first labour and childbirth experience in last 24–48 h will be invited to participate in the study.

Subjects to control ratio of 1:1 will be taken using known-groups validation.

- *Subjects* Women who are nulliparous and undergoing operative deliveries (forceps deliveries, vacuum extractions, emergency caesarean sections).
- *Controls* Women who are nulliparous and undergoing normal vaginal delivery.

### Sample size calculation

CEQ has 22 items in four domains. According to the accepted method of 5 to 10 times the observed variables, subject to variable ratio of 10:1 will be taken [1, 11]. Therefore, a minimum sample size of 220 with 22 items will be calculated. To achieve a 70% response rate as in original Swedish study, it was decided to recruit 350 women to get a final sample of 220 women. Convenience sampling method will be adopted.

### Duration of the study

The study will continue until the relevant numbers are obtained. The duration of the study is approximately 05–06 months based on delivery rate.

### Inclusion and exclusion criteria

#### Inclusion criteria

- Pregnant women who are more than 16 years of age and mothers who have undergone their first labour and childbirth experience in last 24–48 h.
- They should have a singleton live fetus and women who will be laboring at term (including women who will be having a caesarean delivery in their latent phase of labour and also women with failed induction of labour).

#### Exclusion criteria

- Women who will be having a baby who is not alive anymore or women with their babies are getting admitted to a special unit (e.g. Neonatal Special Care Baby Unit or Intensive Care Unit).
- Those who are not willing to participate will be excluded from the study.
- Those who cannot read or write in Sinhala language will be excluded.
- Women who are undergoing planned elective cesarean deliveries.
- Women who are not educated up to G.C.E. Ordinary Level will also be excluded.

### Translation process

The original English language CEQ will be translated into Sinhala language according to the standard validation protocols before its final validation study as recommended by the World Health Organization (WHO) [12]. The linguistic translation process will be carried out involving following basic steps [13]. Literal translation (forward translation) and adaptation of the English questionnaire into Sinhala lifestyle and cultural context will be undertaken by two accredited bilingual native Sinhala speakers. One of them will be a woman in reproductive age. After receiving the both Sinhala translations, these will be compered by the research group at Ragama, Sri Lanka. Discussion with obstetricians, labour room staff and patients will also be carried out as to whether the questionnaire measures what it was designed to measure and that clinically meaningful aspects will not be lacking. With their comments, the Sinhala version of questionnaire will be rephrased according to Sri Lankan cultural context and will be compiled them into one Sinhala language version. Back-translation from Sinhala to English will be performed by a single accredited translator who will also be a bilingual native speaker. Then, review of the back-translation will be carried out by the CEQ team at the Sahlgrenska Academy, University of Gothenburg, Sweden. The final Sinhala CEQ translation will be produced after modifications, re-translation of necessary items to balance the discrepancies during translation process. Validation study will be carried out using this final translation. During the literal translation, the translators will be given the opportunity to give us options if there are different ways to translate any of the items and/or any difficulties in the translation process.

### Study instrument

Sinhala version of the CEQ will be used as the study instrument. The CEQ consists of 22 items referring to the childbirth experience (Table 1). Women’s responses to 19 items in CEQ will be rated on a 4-point Likert scale and remaining 3 items will be evaluated by means of a visual analogue scale (VAS). These VAS scores will then be converted into categorical variables facilitating interpretation and analysis; 0–40 = 1, 41–60 = 2, 61–80 = 3 and 81–100 = 4. Here, negatively worded items will be reversed. All 22 items in CEQ are divided into 4 groups as 4 main domains as mentioned above; “own capacity” (8 items about sense of control, their feelings during birth process and labour pain; 1, 2, 4, 5, 6, 19, 20, 21 items), “professional support” (5 items regarding information and the midwifery care they received; 13, 14, 15, 16, 17 items), “perceived safety” (6 items about sense of security and memories from their labour and childbirth; 3, 7, 8, 9, 18, 22 items), and “participation” (3 items about own possibilities to influence maternal position, their movements and pain relief in labour; 10, 11, 12 items).

**Table 1:** Main four domains and included items in Childbirth Experience Questionnaire (CEQ)

Domain	Items
Own capacity	Labour and birth went as I had expected I felt strong during labour and birth I felt capable during labour and birth I was tired during labour and birth I felt happy during labour and birth I felt that I handled the situation well As a whole, how painful did you feel childbirth was? As a whole, how much control did you feel you had during childbirth?
Professional support	My midwife devoted enough time to me My midwife devoted enough time to my partner My midwife kept me informed about what was happening during labour and birth My midwife understood my needs I felt very well cared for by my midwife
Perceived safety	I felt scared during labour and birth I have many positive memories from childbirth I have many negative memories from childbirth Some of my memories from childbirth make me feel depressed My impression of the team’s medical skills made me feel secure As a whole, how secure did you feel during childbirth?
Participation	I felt I could have a say whether I could be up and about or lie down I felt I could have a say in deciding my birthing position I felt I could have a say in the choice of pain relief

### Data collection procedure

Eligible women will be identified in the postnatal ward by the principal investigator (MP) and will be invited to participate. They will be given the information sheets and consent forms in Sinhala language. Those who will agreed to join recruited by signing an informed written consent form. Women will be received a clear description of the procedure by the investigator. Initially basic demographic and clinical details will be collected into a data collection sheet.

Two copies of CEQ with two stamped-envelopes will be given to each mother and will be asked to fill each in 1 month postnatal and 6 weeks postnatal. Soon after childbirth, mothers are supposed to have gone through their first labour experience and having relatively fresh memories of their childbirth experience. As well as, mothers have minimum contact with their childbirth caregivers at hospital after 4 weeks. Therefore chances of giving socially desirable responses will be minimal and minimizing potential bias. Recruited women will be offered the option of completing the CEQ at home and sending it via post and also to ask for clarifications by making a phone call to the investigator. Reminders will be sent after 1 month and 6 weeks as a phone call or a post card. Demographic details and their delivery details will be collected from the clinical notes and interviewing them.

### Data analysis

Basic demographic characteristics of the data will be presented using descriptive statistics. These will also be analysed to see any deviations. Continuous variables will be presented as means and standard deviations. Pearson Chi Square test will be applied to any difference between categorical variables. Mann- Whitney U test will be applied to compare any skewed continuous variables. P value < 0.05 will be considered as statistically significant.

### Face validity

Face validity will be done for the Sinhala CEQ version to see the items were well understood and easy to answer to. This will be done by giving questionnaire to 25 primiparous mothers to find any difficult questions and asking their comments on questionnaire items. The CEQ will also be given to a group of labour ward staff members at NCTH (n = 10) consisting of three experienced nurse-midwives, three midwives and one medical officer who will also be a female in reproductive age group.

### Internal consistency

This will be evaluated using Cronbach’s coefficient alpha. Usually, Cronbach’s alpha ≥ 0.7 is considered as a satisfactory level of internal consistency.

### Construct validity

Construct validity will be evaluated as follows. The first will be whether the questionnaire was able to differentiate between complicated (operative deliveries) and uncomplicated (normal deliveries) deliveries, and secondly to test questionnaire against the delivery outcome. The method of known-groups validation will be used to measure construct validity. This method can be used to evaluate the ability of the CEQ to distinguish between above known groups within the sample and which were known to differ on known key socio-demographic or clinical variables. The CEQ has shown good construct validity and reliability for assessing childbirth experience between its known groups [1].

A comparison will be done between CEQ subscale scores and overall CEQ score for different key characteristics.

For example,

1. Women who had labour onset; spontaneous versus induced
2. Women with duration of labour; more than 12 h versus less than 12 h
3. Women with augmentation of labour; with oxytocin versus without oxytocin
4. Women who had a; normal delivery versus operative delivery.

Here the overall CEQ score means average of the 4 individual CEQ subscale scores. It is well-documented that oxytocin augmentation of labour with oxytocin, operative delivery (instrumental or caesarean delivery) and long duration of labour (> 12 h) known to cause a negative influence on woman’s childbirth experience [14–16].

If the subscale scores are not normally distributed, non-parametric Mann–Whitney U test will be applied to compare subscale scores between subgroups.

### Test–retest reliability

To assess stability (test–retest reliability) a 2-week test–retest analysis of the sample will be done. This agreement will be further assessed by using the weighted Kappa (k) statistic [17]. To represent a substantial agreement between the two scores between 1 month and 6 weeks questionnaires a value of weighted Kappa between 0.61 and 0.80 will be taken [18].

### Maintenance of data

Data will be entered into a data collection sheet and confidentially stored in an ongoing computer database.

### Plan of presentation of results

Findings will be presented at academic symposia and published in indexed peer reviewed journals.

### Ethical considerations

Eligible patients will be identified and counseled by investigator at each study setting. Before entry into the study the investigator will explain to a potential patient the aims, methods, reasonably anticipated benefits and potential hazards of the study. Patient information sheets will be distributed to eligible patients and an independent medical doctor would be available for a more detailed explanation. After sufficient information, informed written consent will be obtained. Illiterate patients and those who are not willing to participate will be excluded from the study.

We have also taken written permission from the original authors of CEQ, Dencker et al. in The Sahlgrenska Academy, University of Gothenburg, Sweden.

Ethical aspects of this study was reviewed and approved by the Ethical Review Committee, Faculty of Medicine, University of Kelaniya, Ragama, Sri Lanka.

### Work plan and time lines

Work plan can be outlined according to the following steps:

1. Literal translation and adaptation of the questionnaire to the cultural context and lifestyle related to the Sinhala language—2 weeks.
2. Discussion with obstetricians, labour room staff and patients as to whether the questionnaire measures and what it was designed to measure and that clinically meaningful aspects are not lacking—1 week.
3. Back-translation from Sinhala to English—1 week.
4. Review of the back-translation by the CEQ team at the Sahlgrenska Academy, University of Gothenburg, Sweden—4 to 6 weeks.
5. The final Sinhala CEQ translation will be produced after modifications to the translation—1 week.
6. Validation study would then be undertaken using this final translation—6 months to 1 year.

## Data Availability

The data that will be gathered from this study will be available upon a request from the author (MP).

## Abbreviations

CEQ: Childbirth Experience Questionnaire
NICE: National Institute for Health and Clinical Excellence
UK: United Kingdom
NCTH: North Colombo Teaching Hospital
GCE: General Certificate of Education
VAS: visual analogue scale
WHO: World Health Organization

## References

[1] Dencker A, Taft C, Bergqvist L, Lilja H, Berg M (2010). Childbirth experience questionnaire (CEQ): development and evaluation of a multidimensional instrument. BMC Pregnancy Childbirth.

[2] Walker KF, Wilson P, Bugg GJ, Dencker A, Thornton JG (2015). Childbirth experience questionnaire: validating its use in the United Kingdom. BMC Pregnancy Childbirth.

[3] National Institute for Health and Clinical Excellence. Intrapartum care of healthy women and babies; 2014. http://www.nice.org.uk/guidance/cg190. Accessed 05 June 2016.

[4] Cook K, Loomis C (2012). The impact of choice and control on women’s childbirth experiences. J Perinat Educ.

[5] Larkin P, Begley CM, Devane D (2012). ‘Not enough people to look after you’: an exploration of women’s experiences of childbirth in the Republic of Ireland. Midwifery.

[6] Hodnett ED, Gates S, Hofmeyr GJ, Sakala C. Continuous support for women during childbirth. Cochrane Database of Systematic Reviews 2013, Issue 7. Art. No: CD003766. 10.1002/14651858.cd003766. http://www.cochrane.org/CD003766/PREG_continuous-support-for-women-during-childbirth. Accessed 10 June 2016. 10.1002/14651858.CD003766.pub5 23857334

[7] Pang MW, Leung TN, Lau TK, Hang Chung TK (2008). Impact of first childbirth on changes in women’s preference for mode of delivery: follow-up of a longitudinal observational study. Birth.

[8] Patabendige M, Senanayake H (2015). Implementation of the WHO safe childbirth checklist program at a tertiary care setting in Sri Lanka: a developing country experience. BMC Pregnancy Childbirth..

[9] Senanayake HM, Patabendige M, Ramachandran R (2018). Experience with a context-specific modified WHO Safe Childbirth Checklist at two tertiary care settings in Sri Lanka. BMC Pregnancy Childbirth.

[10] Senanayake HM, Patabendige M, Ramachandran R (2018). Piloting of WHO safe childbirth checklist using a modified version in Sri Lanka. BMC Res Notes.

[11] Fayers P, Machin D (2000). Quality of life: assessment, analysis and interpretation.

[12] World Health Organization. Process of translation and adaptation of instruments. Geneva: World Health Organization 2016. https://www.who.int/substance_abuse/research_tools/translation/en/. Accessed 01 Apr 2018.

[13] Wild D (2005). Principles of Good Practice for the Translation and Cultural Adaptation Process for Patient-Reported Outcomes (PRO) Measures: report of the ISPOR Task Force for Translation and Cultural Adaptation. Value Health.

[14] Waldenstrom U, Hildingsson I, Rubertsson C, Radestad I (2004). A negative birth experience: prevalence and risk factors in a national sample. Birth.

[15] Brown S, Lumley J (1998). Changing childbirth: lessons from an Australian survey of 1336 women. Br J Obstet Gynaecol.

[16] Nystedt A, Hogberg U, Lundman B (2005). The negative birth experience of prolonged labour: a case-referent study. J Clin Nurs.

[17] Altman D (1990). Practical statistics for medical research.

[18] Landis JR, Koch GG (1977). Measurement of observer agreement for categorical data. Biometrics..

